# Hybrid soft computing systems for electromyographic signals analysis: a review

**DOI:** 10.1186/1475-925X-13-8

**Published:** 2014-02-03

**Authors:** Hong-Bo Xie, Tianruo Guo, Siwei Bai, Socrates Dokos

**Affiliations:** 1 Graduate School of Biomedical Engineering, University of New South Wales, Sydney 2052, Australia

**Keywords:** Electromyography, Hybrid soft computing system, Pattern classification, Modeling, Neuromuscular disease diagnosis

## Abstract

Electromyographic (EMG) is a bio-signal collected on human skeletal muscle. Analysis of EMG signals has been widely used to detect human movement intent, control various human-machine interfaces, diagnose neuromuscular diseases, and model neuromusculoskeletal system. With the advances of artificial intelligence and soft computing, many sophisticated techniques have been proposed for such purpose. Hybrid soft computing system (HSCS), the integration of these different techniques, aims to further improve the effectiveness, efficiency, and accuracy of EMG analysis. This paper reviews and compares key combinations of neural network, support vector machine, fuzzy logic, evolutionary computing, and swarm intelligence for EMG analysis. Our suggestions on the possible future development of HSCS in EMG analysis are also given in terms of basic soft computing techniques, further combination of these techniques, and their other applications in EMG analysis.

## Background

During a voluntary contraction of skeletal muscles, the electrical activity of activated motor units can be detected with surface or inserted electrodes. The resulting electromyographic (EMG) signal is the summation of motor unit action potentials (MUAPs) discharged by the muscle fibers nearby recording electrodes [1,2]. EMG contains rich information of motor unit recruitment and firing, motion intention, and general physiological state of neuromuscular system. Since EMG directly reflects neuromuscular activities, EMG signal analysis has been applied in prosthetic devices control [3-6], human-machine interaction [7-9], functional electrical stimulation [10-12], kinematic parameters prediction [13-15], and neuromuscular diseases diagnosis [16-18]. The application of EMG varies from medicine, rehabilitation to sports, from biomechanics, ergonomics to basic physiology [19].

One of the main challenges in EMG analysis is the low quality of EMG signals. EMG is not always strictly repeatable, and may sometimes even be contradictory since it may be modified by many factors such as muscular fatigue, electrode shift, sweat, changing in thickness of skins, tissues. Moreover, it is a weak bio-signal contaminated by the noise from the internal cross-talk, ambient electromagnetic radiation, and movement artifacts. The major tasks for EMG analysis can be summarised as pattern detection, classification, decomposition, and modelling. A basic scheme for such analysis often includes a front-end feature extraction and a back-end classification or regression model. In order to extract differentiable features from noisy EMG signals, various features have been exploited in time, frequency, time-frequency, and phase-space domains, most of which have been reviewed by Phinyomark et al. [20]. To identify the general physiological state of neuromuscular system or motion intent from extracted features, many traditional statistical classification or regression models have been suggested to resolve the problem using extracted EMG features [21]. These statistical models include linear discriminant analysis (LDA) [22], quadratic discriminant analysis (QDA) [23], Gaussian mixture models (GMM) [24], hidden Markov models (HMM) [25], and *k*-nearest neighbors classifier [26].

In the meantime, with the rapid advance of soft computing (SC) techniques, a substantial amount of research efforts has been directed to its application in EMG-related pattern classification and regression problems. The term soft computing was first proposed by Zadeh [27] for constructing a new generation of computational intelligent system. The ultimate goal of soft computing is to provide human-like expertness such as specific knowledge for a particular domain, uncertain reasoning, and adaptation to a time varying environment. All these features of soft computing are important for solving practical computing problems. Conventional artificial intelligence techniques only deal with precision and certainty, whilst soft computing, by exploiting the tolerance for imprecision, uncertainty, and partial truth with low solution cost, can achieve tractability, robustness, and better report with reality. Soft computing consists of several techniques, among which artificial neural networks (ANN), evolutionary computing (EC), fuzzy logic (FL), and swarm intelligence (SI) have been the most widely-used in the past few years [28]. However, a consensus to the exact scope or definition of SC has not yet been reached. For instance, swarm intelligence (SI), a family of nature inspired algorithms, has recently emerged as a new branch of SC, which is capable of producing low-cost, fast, and reasonably accurate solutions to complex problems [29-31]. Unlike hard computing methods, SC methods cope up with problems that deal with imprecision, uncertainty, learning, and approximation to achieve tractability, robustness, and low-cost solutions [32]. The unique property of SC is that it is heavily involved in learning from experimental data, making it suitable for EMG analysis. Many methods based on a single technique of ANN, EC, FL, and SI have been evaluated based on their applicability to EMG analysis, some of which can be found in earlier reviews [21,33].

Constituent components of soft computing have provided efficient solution to wide range of problems in different domains including EMG analysis. However, each of these technologies has its inherent advantages and disadvantages. Taking ANN for example, the precision of its output is often limited to least square errors. Its training time is often quite long, and the training data have to be chosen over the entire range where the variables are expected to change. In addition, it is difficult to determine the proper size and structure of an ANN to solve a given problem. As for the fuzzy logic algorithms, the correct set of fuzzy rules and membership functions are difficult to be determined to describe system behaviour, as the system complexity increases. The use of fixed geometric-shaped membership functions in fuzzy logic limits system knowledge more in the rule base than in the membership function base, resulting in requiring more system memory and processing time. Moreover, fuzzy logic utilises heuristic algorithms for defuzzification, rule evaluation, and antecedent processing. However, heuristic algorithms are also problematic since heuristics do not guarantee satisfactory solutions under all possible conditions. Fortunately, the significance of different SC techniques lies in the fact that they are complementary, instead of competitive. In many cases a problem can be solved by combining these techniques rather than using one exclusively. It is therefore appropriate to fuse these techniques into a hybrid soft computing system (HSCS), so that the merits of one technique can compensate for the demerits of another. Moreover, HSCS is beneficial for technique enhancement, multiplicity of application tasks, and realizing multifunctionality [34]. The aim of this paper is to provide a comprehensive review of key applications of various hybrid SC methods used in EMG analysis. Based on the review, we will also present our suggestions for HSCS in EMG analysis in future research.

### Neural-fuzzy hybridisation

Neuro-fuzzy systems have been the most widely exploited HSCS in EMG pattern recognition, which are summarised in Table 1. In order to discriminate 10 hand/wrist motions from 16-channel EMG signals, Khushaba and Al-Jumaily [35] used fuzzy C-means (FCM) to select those wavelet features that maximised the class separability. Principal component analysis (PCA) was then employed to remove redundancy by projecting the features onto their eigenvectors. Finally, the resultant features were fed to a multilayer perception (MLP) to be classified into different patterns. FCM was subsequently replaced by fuzzy entropy measure to determine features suitability in classifying the same EMG datasets [35]. Both fuzzy logic based feature selection techniques combined with MLP could produce about 99% classification accuracy by using only a small portion of the original feature set. Malcolm and Granat [36] applied a neuro-fuzzy classifier to classify single-site EMG signals from the biceps and triceps brachii muscles. The aim was to detect the intention of a paraplegic person to stand up or to sit down for use with an electrical stimulation orthosis. This neuro-fuzzy hybridisation was functionally based on the Surgeno-type fuzzy rule base; at the same time, it had an architecture equivalent to a radial basis function (RBF) neural network under some constraints, allowing the system to learn from the training data. This neuro-fuzzy classifier was found capable of identifying 29 standing and 28 sitting EMG signals out of 60 EMG signals by using seven bell-shape membership function and 30 rules. Balbinot and Favieiro [37] evaluated the performance of a similar Sugeno fuzzy model-based adaptive neuro-fuzzy system to classify seven distinct movements in a long test duration lasting for about three hours, achieving an average accuracy of 86%. Karlik et al. [38] presented a comparative study of classification accuracy of EMG signals using the MLP, back-propagation (BP) network, conic section function NN, and fuzzy clustering neural network (FCNN). In this study, a fuzzy clustering-based approach was first adopted to compute autoregressive (AR) model parameters and their signal power before these values were fed to ANN. After the test, the recognition rates using FCNN varied between 95% and 100%, with average recognition accuracy of 98%.

**Table 1:** A summary of hybrid neural-fuzzy techniques applied to EMG analysis

**Reference**	**Task**	**Techniques**	**Results**
[35]	Classification: 10 hand motions	Fuzzy-C means + MLP	Accuracy: 99%
[35]	Classification: hand motions	Fuzzy entropy + MLP	Accuracy: 99%
[36]	Classification: 2 leg motions	Surgeno model + RBF	Accuracy: 57/60
[37]	Classification: 7 arm motions	Surgeno model + MLP	Accuracy: 86%
[38]	Classification: 6 hand motions	Fuzzy clustering NN	Accuracy: 95% ~ 100%
[39-41]	Classification: 6 classes hand motions	ANFIS	Accuracy: 96.7±1.2% [40] Accuracy: maximum 100%, mean 92% [41] Accuracy: mean 94% [42]
[42]	Prediction gait events	ANFIS	Accuracy: 95.3%. ~ 98.6%.
[43,44]	Classification: 3 arm motions	Abe-Lan fuzzy network	Abe-Lan fuzzy network performed better than SOM, FCM, and MLP
[45]	Classification: 6 motions	Fuzzy Min-Max ANN	Accuracy: 10% higher than without fatigue compensation
[46]	Classification: 4 hand motions	Fuzzy SVM	Accuracy: Fuzzy SVM outperformed BP NN 5%
[47]	Classification: 6 hand motions	FuzzyEn + ELM, CrEn + ELM	CrEn outperformed FuzzyEn
[48]	Classification: 10 grasps or in-hand manipulations	FGMM	Accuracy: 96.7% of FGMM, better than HMM, SVM
[49,50]	Modelling: EMG-movements	ANFIS	Accuracy: 97%, 99%, 87.9%, and 81.8% for four subjects, respectively
[51]	Modelling: force moment and velocity-peak EMG	Neuro-fuzzy	Error: 4.97% ~ 13.16%
[52,53]	Modelling: kinematics-EMG-force	RFNN	Small prediction error
[54]	Modelling: EMG-force moment	Takagi-Sugeno	EMG-to-activation model performed better than Takagi-Sugeno
[55,56]	Control upper-limb exoskeleton	Neuro-fuzzy	Effectiveness of the control method
[7]	Control upper-limb exoskeleton	Neuro-fuzzy	Low RMS errors
[57]	Control ankle exoskeleton	Neuro-fuzzy	Low RMS errors
[58]	Diagnosis	Fuzzy integral + BP NN	Accuracy: 80.95±7.2%
[16]	Diagnosis	FSVM	Accuracy: 93.5±1.4% FSVM performed better than LDA, BP and RBF
[59]	Decomposition	AFNNC	Accuracy: AFNNC performed better than ACC at roughly 6.1%
[60]	Diagnosis	NEFCLASS	Accuracy: 90%
[61]	Diagnosis	ANFIS	Accuracy: 76.43%

Adaptive neuro fuzzy inference system (ANFIS) is a kind of neural network structure based on Takagi–Sugeno fuzzy inference system. Since it integrates both neural networks and fuzzy logic principles, it has the potential to capture the benefits of both techniques in a single framework. Its inference system corresponds to a set of fuzzy IF–THEN rules, which is capable of learning to approximate any nonlinear functions. Khezri and Jahed [39] integrated the ANFIS with a real-time learning scheme to identify six classes of hand motion commands from EMG signals. In their work, a hybrid method, consisting of back-propagation and least mean square (LMS), was utilized to train the fuzzy system. In addition, in order to optimize the number of fuzzy rules, a subtractive clustering algorithm based on a measure of data point density in the feature space was developed. The idea behind this approach was to find regions in the feature space with high densities of data points. Their results revealed that the real-time ANFIS approach provided 96.7±1.2% average accuracy, superior to the BP artificial network (87.3±2.6%). Furthermore, they compared the effects of EMG features in time and time–frequency domains respectively and their combination [40]. The recognition scheme utilizing the combined features with an ANFIS classification provided the best result in identifying the same six classes of hand movements. With a maximal identification rate of 100% and an average classification accuracy of 92%, the ANFIS system proved outstanding in comparison with other studies using relevant algorithms such as ANN, LDA, Bayes’ classifier, fuzzy interference system, and nonlinear discriminant functions. Zhang et al. [41] also designed a similar neuro-fuzzy system with C-means algorithm for initialising fuzzy membership functions to classify six hand motions. The feature set they used was singular values of a wavelet coefficient matrix. The accuracy of their approach was higher than MLP by roughly 6% across six motions. ANFIS with a supervisory control system (SCS) was also used to predict the occurrence of gait events by analysing the EMG activity of lower extremity muscles in children with cerebral palsy (CP) [42]. In this study, a Type-3 ANFIS, i.e., an ANFIS employing a first-order Sugeno fuzzy model, was employed as the rule generator [62]. Using one EMG signal and its derivative from each leg as inputs, the ANFIS with SCS was able to predict all gait events in seven out of eight children. Overall accuracy in predicting gait events ranged from 98.6% to 95.3%.

Some more sophisticated neuro-fuzzy systems were also developed to classify complicated EMG patterns. Micera et al. [43] utilised a hybrid supervised learning scheme termed Abe-Lan fuzzy network to EMG pattern analysis for classification of arm movements. The method required that the input feature space was subdivided into a number of regions, i.e., hyperboxes. The second-layer units consisted of fuzzy rules, which calculate the degrees of membership for the rules to resolve the overlaps between activation hyperboxes. The third-layer units for the *i*th class took the maximum value of the inputs from the second layer; the fourth-layer units took the minimum value among the maximum values generated by the third layer. Finally, an unknown feature vector was recognised as the class with the maximum membership assignments. The study found the classification method able to correctly classify all the EMG patterns related to the selected planar arm pointing movements. In a follow–up comparative study, Micera et al. [44] compared self-organizing map (SOM), fuzzy c-means (FCM), and MLP, to the Abe-Lan fuzzy network in classifying those three muscles in the same arm pointing task with small-size training sets. The results indicated that Abe-Lan fuzzy network was more robust than SOM, FCM, and MLP when working with small training sets. Muscular fatigue causes decrease in recognition rates due to the time-varying EMG feature, and therefore Song et al [45] adopted a Fuzzy Min-Max neural network (FMMNN) to compensate for the fatigue effect for robust EMG classification. In FMMNN, each fuzzy set is a union of fuzzy set hyperboxes. Fuzzy set hyperbox is an *n*-dimensional box defined by a min point and a max point with a corresponding membership function. Since EMG feature variations during muscle contractions are consistent, dynamically adjusting min-max values of hyperboxes according to the contraction time in FMMNN learning could compensate for the fatigue effect. The method was evaluated to follow four-channel EMG signals of six motions, and a significant improvement in recognition rates was found.

Support vector machine (SVM) is another machine learning method based on statistical learning theory, more suitable for small sample classification problems [63]. However, SVM was originally developed for solving regression and binary classification problems [46]. Several techniques have been proposed to extend a binary classifier to multi-class problems, including one-against-all (OAA), one-against-one (OAO, also known as pairwise), and error-correcting -output code (ECOC) [64]. However, indecisive regions often exist in these strategies to classify multiple patterns [46]. In order to avoid the indecisive regions, Yan et al. [46] constructed a fuzzy support vector machine (FSVM) model, in which a fuzzy membership function was utilised to transfer the output of a SVM discriminant function into a fuzzy class score. They made a comparison between the FSVM scheme and a BP neural network. The results indicated that four hand/wrist motions could be identified by FSVM with about 5% higher accuracy than BP network. Fuzzy entropy (FuzzyEn) is an improved nonlinear time series complexity measure which utilizes a continuous and convex fuzzy membership function to quantify the similarity between vectors’ [65]. Shi et al. [47] compared EMG classification performance by features extracted from FuzzyEn, approximate entropy (ApEn), sample entropy (SampEn), and cumulative residual entropy (CrEn). Classifiers compared in the study were SVM, LDA, single-hidden layer NN, and extreme learning machine (ELM). They suggested the combination of CrEn and ELM, a new NN architecture, performed significantly faster than other hybrid systems in EMG pattern decision. In order to equip Gaussian mixture models with nonlinear fitting capabilities, Ju et al. proposed a fuzzy Gaussian mixture model (FGMM) [48]. They found that FGMMs outperformed commonly used approaches including LDA, HMM, and SVM in terms of the accuracy in recognising different ten hand grasps and in-hand manipulations captured from different subjects. The best performance with the recognition rate of 96.7% was achieved by FGMMs with the multi-feature combining Willison amplitude and Determinism extracted from recurrence qualification analysis (RQA).

Apart from EMG pattern recognition, HSCS is often adopted to model the relationship between EMG signals and kinetics or kinematics variables. Liu and Young [49] proposed an initial point detection method to establish the relationship between the upper-arm EMG signals and corresponding movements, avoiding the learning and training processes accompanied by huge computational loads and complicated system. Via ANFIS, the critical values for the initial point detection method adopted in the proposed system were determined. In a subsequent study, Liu and Young [50] used the empirical mode decomposition (EMD) method to break down the EMG signals into a set of intrinsic mode functions (IMF) to replace time domain features in their previous study [49], with each IMF representing different physical characteristics of muscular movement. ANFIS they employed in the study was not intended to provide a classifier could accurately identify human intention from EMG signals, but to efficiently establish the relationship between EMG signals and corresponding movements. The prediction accuracy for four subjects was 97%, 99%, 87.9%, and 81.8%, respectively. Lee et al. [51] developed a neuro-fuzzy model to predict peak EMG values for trunk muscles based on lifting task variables. Their model utilised two task variables, trunk moment and trunk velocity, as inputs, and 10 types of muscle activities as outputs. The input and output variables were represented using the triangular-shape membership functions. The initial fuzzy rules were generated by a vector quantization neural network using collected EMG data. The vector quantisation algorithm included the differential competitive learning rule that combined competitive and differential Hebbian learning. The final fuzzy rules were used to derive the prediction model. The developed model was capable of estimating the normalised peak EMG values only with the mean absolute error ranging from 4.97% to 13.16%. Since it is difficult to directly evaluate the spinal forces from kinematics, Hou et al. extended the above work to establish the kinematics-EMG-force relationship and modelled the dynamics of muscular activities based on a four-layer recurrent fuzzy neural network (RFNN) [52]. EMG signals were used as an intermediate output and were fed back to the input layer. The trained model could then have the forces predicted directly from kinematic variables while bypassing the procedure of measuring EMG signals and avoiding the use of biomechanics model. Karwowski et al. [53] further improved the above kinematics-EMG prediction model in two aspects: they modelled the EMG responses for 10 trunk muscles in manual-lifting tasks using new fuzzy relational rule network (FRRN) architecture. FRRN was a modification of a conventional fuzzy rule based hybrid neuro-fuzzy system which, in addition to modelling the input-output relationship, allows linear relationships among the input variables of the system to be modelled with reduced complexity. Brzostowski and Swiatek [54] modelled the relationship between EMG signals and the force moment generated by moving upper or lower limb using a hybrid method termed Takagi-Sugeno system. EMG-to-activation model, ARMAX model, Box–Jenkins model, and a neural network were also conducted for comparison.

Variables predicted by or combined with EMG have also been used to drive various external devices. Kiguchi et al. [55] developed robotic exoskeletons controlled by EMG signals using a neuro-fuzzy algorithm to assist motion of physically weak persons. In their method, two kinds of nonlinear functions were applied to express the membership function of the neuro-fuzzy controller. The initial fuzzy IF–THEN control rules were designed according to the human elbow and shoulder motion patterns analysed in the pre-experiment, and then transferred to the neural network form. The input variables for the neuro-fuzzy controller were eight kinds of mean absolutes of EMG, with 21 rules in each neuro-fuzzy controller. The outputs of the neuro-fuzzy controller were the torque command for shoulder motion, desired impedance parameters, and desired angle for elbow motion of the exoskeleton system. The effectiveness of proposed control method in human upper-limb motion assist was validated by the trajectory of the wrist defined by a compound sine and cosine function on the horizontal plane. Subsequently, they improved their neuro-fuzzy controller to five layers: the input, fuzzifier, rule, defuzzifier, and output layer, and the number of EMG channels increased from eight to twelve [56]. In order to solve the problem of EMG-based control caused by human anatomy, multiple neuro-fuzzy controllers were applied in this system. When the magnitude of the muscle activity levels of the user was small, the exoskeleton was controlled based on the wrist force sensor to avoid mis-operation. When the user activated the muscles, the force sensor signals were ignored and the EMG-based control was evolved. Kiguchi and Hayashi [7] recently designed an improved upper-limb power-assist exoskeleton using a 16-channel EMG impedance control method. A five-layer neuro-fuzzy muscle-model matrix modifier was applied to take into account the effect resulting from the difference of user upper-limb posture. Input variables to the neuro-fuzzy modifier were joint angles of theupper limb, whilst the modifier output the coefficient for each weight of the muscle-model matrix to modify the weight matrix in real time based on the upper-limb posture. A neuro-fuzzy controller integrating EMG sensor and artificial proprioceptor, which imitated the closed-loop control system of human body, was developed to achieve real-time control of the ankle exoskeleton by Fan et al. [57]. Their controller combined fuzzy rules established on detailed anatomical knowledge and the results of previously performed experiment with hybrid learning algorithm. It was built to decode the human motion in real time using the fusion of EMG signals and the precise proprioception providing joint angular information feedback. Corresponding experimental results demonstrated that the parallel ankle exoskeleton met the kinematical and dynamical requirements of ankle joint, and the neuro-fuzzy controller with proprioception was accurate and effective with low root mean square (RMS) errors.

EMG is the summation of motor unit action potential (MUAP) trains from all active motor units within the recording electrode range. MUAPs provide important information for assessing neuromuscular disorders. Xie et al. [58] presented a hybrid decision support system based on the fusion of outputs from multiple neural networks using fuzzy integral to enhance the diagnosis accuracy. BP network was used as a single diagnosis model in three various feature domains, i.e., morphological measures, frequency parameters, and wavelet transform features. The outputs of three BP networks were combined using fuzzy integral to provide a final diagnosis evaluation. The consensus diagnosis obtained from fuzzy integral achieved 80.95 ± 7.2% accuracy, higher than each single network in discriminating patients with motor neuron disease or myopathic patients from normal subjects. Subasi [16] conducted a similar experiment to discriminate neurogenic or myopathic subjects based on MUAPs analysis, achieving 93.5±1.4% diagnosis accuracy. The comparative analysis suggested that FSVM was superior to LDA, BP and RBF network, C4.5 decision tree, and SVM in at least three aspects: moderately high recognition rate; insensitivity to overtraining; and consistent outputs which demonstrated high reliability. Decomposition of surface or intramuscular EMG signals into their constituent MUAPs is the primary step before MUAPs based neuromuscular diagnosis. Rasheed et al. [59] presented an adaptive fuzzy *k*-nearest neighbour classifier (AFNNC) for EMG signal decomposition. The performance of the developed classifier was compared to an adaptive template matching classifier, an adaptive certainty classifier (ACC), by using synthetic signals with specific properties and experimental signals. For experimental EMG signals, the AFNNC had on average an improved correct classification rate (8.1%) compared to ACC. Others tried to diagnose neuromuscular disorders by analysing surface EMG signals without decomposition. Kocer [60] fed AR model coefficients of EMG signals into a three-layer neuro-fuzzy system termed NEFCLASS, and 90% classification success rate was obtained from this system using EMG signals collected from 177 subjects. Compared with MUAPs analysis, their study suggested that the application of AR model coefficients of EMG signals followed by the neuro-fuzzy system might produce a new and reliable classification system for rapid diagnosis. Khasawneh et al. [61] collected three types of electrophysiological signals including EMG, electroencephalogram (EEG), and electrooculogram (EOG) to train an ANFIS system for sleep multistage level scoring. The input pattern adopted to train the ANFIS subsystem was a set of extracted features based on the entropy. Finally, an output selection subsystem was utilized to provide an appropriate sleep stage according to the ANFIS stage subsystems outputs. The developed system was able to provide an acceptable estimation for six sleep stages with an average accuracy of 76.43%. In order to improve the medical information management and decision making, Arasu and Palanisamy [66] proposed the concept of neuro-fuzzy agents (NFA) to analyse the electrophysiological signals, such as EEG, ECG, and EMG, recorded from different patients located across the hospital campuses.

### Neural-evolutionary hybridisation

Evolutionary computing conventionally refers to four main algorithms: genetic algorithms (GA), genetic programming (GP), evolutionary strategies (ES), and evolutionary programming (EP) [67]. More recent developments, such as gene expression programming (GEP) [68], cultural algorithm (CA) [69], and differential evolution (DE) [70], obtain some characteristics of the earlier algorithms. In hybrid neural-evolutionary methods, evolutionary computing techniques are mainly adopted to select discriminant features from the feature pool, and to optimize the structure or parameters of a neural network for EMG classification or modelling, all of which are summarised in Table 2.

**Table 2:** A summary of hybrid neural-evolutionary techniques applied to EMG analysis

**Reference**	**Task**	**Techniques**	**Results**
[71]	Classification: 6 arm motions	GA + MLP + HMMs	Accuracy: 87.7%
[72,73]	Classification: 7 wrist motions	GA + BP ANN	Accuracy: 5% ~ 10% [74,75], 10% [76] improvement compared to without GA optimization
[74]	Classification: 7 wrist motions	GA + MLP	Feature reduction rate: 70% Accuracy: 6% improvement
[75]	Classification: 6 hand motions	GA + MLP	Error: 4.89%
[76]	Classification: 4 hand motions	GA + BP ANN	Accuracy: 91.38%
[77]	Classification: 4 hand motions	GA + RBF + MLP	Accuracy: 6% improvement compared to MLP
[78]	Classification: 4 hand motions	GA + FFT + PCA + MLP	Improved accuracy and speed
[79]	Modelling: EMG-on/off signals during stride	GA + BP ANN	Improved speed
[80]	Classification: 12 finger motions	GA + SVM	Reducing 8 ~ 11 channels and comparable accuracy
[81]	Classification: 10 hand motions	GA + BP ANN	Accuracy: 98%
[82]	Classification: 6 wrist motions	GA + RBF	Accuracy: 75%
[83]	Classification: 2 muscle states	GA + SVM	Accuracy: 97.3%
[84]	Modelling: EMG-force	GA + BP ANN	Accuracy: 99%
[85]	Diagnosis	GBLS	Accuracy: 95% for training, 70% for test

In the early 1990s, Kwon et al. [71] described an approach for classifying EMG signals of six arm movements using a MLP with GA and HMMs hybrid classifier. Instead of using MLP as probability generators for HMMs, they utilised MLP with GA as the secondary classifiers to increase discrimination rates of myoelectric patterns. In this study, GA was used to generate network's initial connection weights to shorten the learning time which produced 87.7% recognition rate. Yazama et al. [72] selected essential bands of EMG signals to construct feature vectors using GA. An EMG recognition experiment of seven wrist operations was performed using a BP neural network with the selected frequency band. They subsequently improved their study by maximizing distances of feature vectors between various classes and minimising the variance in a class during GA optimization [72]. There was about 5% to 10% rate improvement using the optimized feature vectors in each person. In the meantime, Yazama et al. [86] also decomposed the original EMG signals into the product of two matrices using non-negative matrix factorization. Noise rejection was then performed by applying a filter optimised by GA to the decomposed matrix. The noise rejection with GA-based filter led to about 10% improved accuracy in a subsequent BP network recognition system. In 2006, Tohi et al. [74] proposed an EMG recognition system that utilised the combination of GA and MLP at two stages to classify seven wrist motions: the extraction of important frequency bands from each signal was performed using the first combination of GA and MLP; a function converting the frequency spectrum into a feature vector was then obtained by using GA and MLP again. GA was applied twice to each subject, and the feature vector specialized in the individual was extracted. The feature reduction rate was about 70% while recognition rate improved about 6% across three subjects. Almost at the same time, Oskoei and Hu [75] adopted a cascaded genetic algorithm as a search strategy to select optimal subset of features from EMG in both time and frequency domains using GA. Davies-Bouldin index (DBI) and Fishers linear discriminant index (FLDI) were employed as the filter objective functions and LDA has been used as the wrapper objective function. 4.89% error rate was obtained for the elite subset of features applied to ANN. Wang et al. [76] utilised similar procedures of GA selected features and a BP network as the classifier to classify EMG signals of four hand motions. Different from Oskoei and Hu [75], Wang et al. used the discrete harmonic wavelet packet transform as the feature extractor to decompose EMG into time-frequency plane, achieving averaged 91.38% accuracy across ten subjects.

Many studies focused on optimising the structure of various EMG classifier or predictor. In 2000, Zabala and Chaiyaratana [77] introduced a hybrid neural structure using RBF and MLP networks. The hybrid network was composed of one RBF network and a number of MLPs, trained by a GA/unsupervised/supervised-combined learning algorithm. The GA and unsupervised learning algorithms were used to locate the centres of the RBF in the hybrid network. In addition, the supervised learning algorithm, based on the BP algorithm, was used to train the connection weights of the MLP in the hybrid network. Their results suggested that the classification accuracy of the hybrid RBF-MLP network was approximately 6% higher than a MLP in the classification four-class EMG signals. Matsumura et al. [78] aimed at constructing a high-speed and high-accuracy EMG recognition system with fast Fourier transform (FFT) for feature extraction, simple-PCA for feature compression, and ANN for recognition. In the meantime, they reduced the node number in the input layer of the ANN using GA optimization to improve training and testing efficiency. According to their simulations, this approach was effective for improving both recognition accuracy and speed. In Chen et al.’s study [79] about a control concept of above-knee prosthesis, surface EMG signals extracted from leg muscles were translated into on-off signal of self-lock control by a hybrid neural network-genetic algorithm to recognize the phase of stride. In their study, GA was applied to avoid the BP network trapping to a local optimum and to speed up the convergence in searching optimal weights. In 2011, Kanitz et al. [80] tested the performance of both channel and feature reduction using GA for EMG data collected from 16 channels on five unimpaired subjects and one transradial amputee performing 12 individual finger movements and a rest state. The classification was performed by a LDA, k-NN, and SVM classifier, respectively. The GA-based optimisation demonstrated a high redundancy in the recorded 16-channel data, as well as an insignificance of certain features — reducing 8 ~ 11 channels, depending on the subject, had little to no effect on the classification accuracy. In the same year, Karimi [81] employed GA to determine the best values for mother wavelet function, decomposition level of wavelet packet analysis, and number of neurons in the hidden layer of BP network in order to obtain a high-speed and small-size BP network structure for EMG classification. This optimised network with minimised size can recognise ten hand motions with a recognition accuracy of over 98%, and it also resulted in an improvement of system stability and reliability for practical considerations. Different from above study, an RBF network was utilised as an EMG recognizer in [82], in which hidden nodes number, centre position, and standardisation parameters were optimised by GA. This GA-RBF structure could reach approximately 75% accuracy for recognising six wrist motions, as well more than or equal to 90% for four motions. Recently, Rong et al. [83] investigated the recognition result of EMG recorded under conditions of a maximum voluntary contraction (MVС) and fatigue states using wavelet packet transform and energy analysis. They used GA to optimise the error penalty parameter and kernel parameters of a SVM classifier. The classification correct rate reached 97.3% with seven fold cross-validation.

Naeem and Xiong [84] proposed a neural-genetic model to predict muscle force from EMG signal, consisting of the genetic state and neural stage. GA was initially employed to convert the raw EMG signal into a representation of muscle activation. At the second stage, the BP neural network method was applied to extract muscle force from the muscle activation obtained previously. Their results showed that the regression of this neural-genetic model exceeded 99%.

Pattichis and Schizas [85] introduced a hybrid diagnosis system combining a neural network and a genetics-based learning system (GBLS) model to classify MUAPs into motor neuron disease and myopathy. The performance of the classifier system was enhanced by updating rules throughout the call of GA, achieving a diagnostic yield higher than 95% and 70% for the training and evaluation sets, respectively.

### Neural-swarm intelligence hybridisation

Swarm Intelligence (SI) has recently emerged as a family of nature-inspired algorithms that are capable of producing low-cost, fast, and reasonably accurate solutions to complex problems [31,87,88]. These algorithms are motivated by the collective social behavior of a group of unsophisticated organisms, such as, ants, bees, birds, and fishes. Although these organisms have very limited individual capability, they can cooperatively interact together to perform complex tasks essential for their survival. The most two popular SI-based algorithms are ant colony optimisation (ACO) and particle swarm optimisation (PSO). ACO draws inspiration from the social behavior of ant colonies, and it has been found both robust and versatile in handling a wide range of optimisation problems [89,90]. PSO is inspired by the social behavior of birds within a flock, in which particles are conceptual entities that fly through the multi-dimensional search space. Unlike most soft computing techniques, PSO does not need the gradient information of objective function. Due to this simplicity, the main strength of PSO is its fast convergence, which compares favorably with other global optimisation algorithms including GA and simulated annealing [91,92]. Table 3 is the summarisation of major findings of hybrid neural-swarm intelligence in EMG analysis.

**Table 3:** A summary of hybrid neural-swarm intelligence techniques applied to EMG analysis

**Reference**	**Task**	**Techniques**	**Results**
[93]	Classification: 8 hand motions	ACO + BP	Better than PCA + BP
[94]	Classification: 10 hand motions	PSO + BP	Accuracy: 99%
[95]	Classification: 6 hand motions	PSO + EMD + PNN	Accuracy: 93.3%
[96]	Classification: 10 hand motions	PSO + MLP	Accuracy: 95%
[97]	Diagnosis	PSO + RBF	Accuracy: 88.92%
[98]	Diagnosis	PSO + SVM	Accuracy: 97.41%

In 2011, Huang [93] proposed an ACO-based feature selection scheme using the heuristic information measured by the minimum redundancy maximum relevance (mRMR) criterion to classify hand motion EMG signals. The mRMR criterion is a fast and accurate feature selection method. Huang used it to approximate the heuristic value of each feature to decrease the computational complexity of ACO searching. His experiments were conducted on ten subjects with eight upper limb motions and two feature sets, i.e., time domain and wavelet transform features. The classification accuracy of using ACO reduced features and BP or LDA was significantly higher than PCA-based method. Khushaba and Al-Jumaily [94] employed PSO to search both the feature and channel space for important subsets. These important subsets were then evaluated to classify an EMG dataset consisting of ten motions associated with three degrees of freedom of the wrist, two different hand grips, and a rest state using a BP classifier. 99% accuracy was achieved for the problem using the PSO method with only 25 features. Shang et al. [95] used the independent component analysis method to eliminate the power frequency interference in EMG. The filtered EMG was then decomposed by EMD. Afterwards, AR model coefficients of IMFs were fed to a probabilistic neural network (PNN) whose transmission rate was optimised by PSO to classify six types of forearm motions. The recognition rate is 93.3% and 85% for with and without EMD, respectively. Ortiz-Catalan et al. [96] made a comparative study of biologically-inspired algorithms to identify different components of EMG patterns upon the control of robotic prosthetics. In order to contribute to a different training paradigm, GA and PSO algorithms were adopted to find the optimal weights of a MLP during training. In addition, since the optimal input set of signal features was unknown at the beginning, an extra GA was used to search for the optimal features, which allowed the fastest training with the most accurate prediction and lowest failure rate. PSO achieved better performance than GA as a training algorithm, providing over 95% accuracy in predicting ten movements. Wu et al. [97] aimed to accurately recognize and predict the tremor onset in the patients with Parkinson's disease and thus implemented an on-demand stimulator. In their approach, by using an RBF neural network based on PSO with local field potential (LFP) data recorded via the stimulation electrodes, the activity related to tremor onset could be predicted. To validate the performance of this hybrid system, EMG signals from patient's forearm were recorded in parallel with LFPs to accurately determine occurrences of tremor. The centres of neurons in RBF neural network were initially determined by a FCM clustering approach, subsequently, the centres and widths were optimized by PSO so that more suitable parameters for the RBF neural network could be found, thus reducing the number of neurons in the hidden layer. Compared to 89.91% detection rate of conventional RBF neural network, this approach demonstrated a comparable detection rate (88.92%) but notable reduction in computational overhead. Subasi [98] proposed a PSO-SVM model that hybridised the PSO and SVM to improve the EMG signal classification accuracy for neuromuscular diagnosis. The penalty parameter and kernel function parameter for the RBF kernel was tuned by PSO. The experiments were conducted to classify EMG signals into normal, neurogenic or myopathic. The PSO-SVM yielded an overall accuracy of 97.41% on 1200 EMG signals selected from 27 subject records, in comparison with 96.75%, 95.17% and 94.08% for the SVM, k-NN, and RBF classifier, respectively.

### Other hybrid soft computing systems

Apart from the approaches reviewed above, many other HSCSs have also been proposed for EMG analysis over the past few years, which are summarised in Table 4. Although ACO was proved to be a powerful technique in different optimisation problems, it still needs some improvements when applied to the feature selection problem. This is due to the fact that ACO builds its solutions sequentially, where in feature selection this behavior will most likely not lead to the optimal solution. Aiming to overcome this problem in EMG classification, Khushaba et al. [99] presented a feature selection algorithm based on a combination of artificial ant and differential evolution (ANTDE) algorithm. The proposed combination enhanced both the exploration and exploitation capabilities of search procedure. The performance of the proposed algorithm was compared with uncorrelated linear discriminant analysis (ULDA) and PCA in a classification task of seven limb motions. The accuracy achieved by the proposed ANTDE was 94.73%, compared to 93.35% and 91.11% for ULDA and PCA respectively for the validation set, while 93.39% for ANTDE against 92.41% and 89.52% for ULDA and PCA respectively for the testing set. Khushaba et al. [100] then proposed another feature projection technique based on a combination of fisher linear discriminant analysis, fuzzy logic, and differential evolution optimisation technique (DEFLDA). This technique assigned different membership degrees to data points in order to reduce the effect of overlapping points in the discrimination process. Furthermore, an optimizing weighting scheme was presented in which certain weights were assigned to the features according to their contribution in the discrimination process. The proposed DEFLDA was tested on publicly available UCI datasets and the same EMG dataset used in [99]. DEFLDA achieved 93.75% and 94.71% accuracies across thirty subjects for time domain and wavelet feature respectively, slightly higher than ANTDE hybridisation. Similarly, a hybridisation of PSO, FL and LDA (PSOFLDA) was developed as an EMG feature reduction technique to reduce the computational cost and enhance the generalisation capability of the classifier [101]. PSO was employed to optimise the weights of features in this approach. The accuracy of this method was similar to ANTDE, but was higher than the conventional FLDA, ULDA, OLDA, and PCA techniques when using the same EMG dataset in [99,101].

**Table 4:** A summary of other hybrid soft computing techniques applied to EMG analysis

**Reference**	**Task**	**Techniques**	**Results**
[99]	Classification: 7 limb motions	ACO + DE	Accuracy: 94.73% (validation), 93.39% (test), better than ULDA and PCA
[100]	Classification: 7 limb motions	FL + LDA + DE	Accuracy: 93.75% (time domain feature), 94.71% (wavelet feature)
[101]	Classification: 7 limb motions	FL + LDA + PSO	Accuracy: Similar to ANTDE in [102], better than FLDA, ULDA, OLDA, and PCA
[102]	Modelling: EMG-force	GA + FL	RMS error: 12.4%

In order to build a model for mapping EMG signals to the force generated by human arm muscles, Rahatabad et al. [102] applied a fuzzy system which was robust to noise and able to model the uncertainties of the muscle. Three fuzzy coefficients were added to the relationships of force-length (active and passive) and force–velocity existing in Hill’s model. Then, a genetic algorithm was used to optimise model parameters to achieve the optimal fit. The proposed fuzzy genetic implementation Hill-based muscle (FGIHM) model had 12.4% RMS error (in a worst case) in comparison to the experimental data recorded from three healthy male subjects. Moreover, the FGIHM active force–length relationship, which was the key characteristics of muscle, was compared to virtual muscle (VM) and Zajac muscle model. The sensitivity of FGIHM was evaluated by adding a white noise with zero mean to the input and FGIHM was proved with lower sensitivity to input noise than the traditional Hill’s muscle model.

## Discussion and conclusion

We have reviewed recent advances of hybrid SC techniques and evaluated their performance on EMG classification, modelling and diagnosis. Most of these studies demonstrated that a hybrid system is able to improve the classification or diagnosis accuracy, robustness to noise, and to speed up the convergence of the system in comparison with a single method. Nevertheless, one major drawback of some HSCSs is the significant increase in the requirement of computational resources. In addition, some HSCSs depend on auxiliary parameters, variables, or structure optimised by its sub-systems or modules to enhance the performance. By summarising the merits and drawbacks of HSCS in present EMG applications, we try to give some personal opinions on the possible future researches of HSCS and its applications in EMG analysis from three perspectives:

### Basic methodology development

Although a variety of hybrid SC methods have been designed and successfully utilised to solve several problems in EMG analysis, they have been mainly limited to neural-fuzzy or neural-evolutionary hybridisation. However, various new types of SC techniques have been developed in recent years. For instance, as the generalisation of the ordinary fuzzy sets, type-2 fuzzy sets are now well established [103,104]. Recent studies have demonstrated that type-2 fuzzy sets significantly outperformed ordinary fuzzy sets in approximation, control, decision making, and clustering. In addition, many new natural or bio-inspired SC methods including artificial immune system, artificial bee colony optimisation, membrane computing, and biogeography-based optimisation, have been proposed recently, presenting various advantages in solving complex problems when co-operated with other SC approaches [105,106]. All of these new SC techniques have provided increased opportunities to develop new and powerful HSCSs.

### Combination

Each SC method has its inherent disadvantages which in return limit its application. The combination or coupling manner is a key factor to design an HSCS. Most existed HSCSs are problem-dependent, and therefore it is hard to provide a generic combination scheme. However, in order to maximise the advantage of each approach, an auxiliary or embedded combination is more favourable than a simple sequential grouping. Other signal processing theories or methods, such as belief function and evidence reasoning [107], may also be applied to effectively integrate various SC methods.

### Applications

Present applications of HSCS are mainly limited to EMG pattern recognition, neuromuscular disease diagnosis, and EMG-force/torque modelling. There still are many other important issues in EMG-based studies in which serious challenges and difficulties exist. HSCS can thus be regarded as a promising way in future to solve these problems including differentiating MUAP waveforms in surface EMG recorded in high voluntary contraction level, modelling multi-scale electric-mechanical relationship in skeletal muscles, reducing channel and feature of high density EMG signals, and identifying muscle synergies.

## Abbreviations

ACC: Adaptive certainty classifier; ACO: Ant colony optimization; AFNNC: Adaptive fuzzy *k*-nearest neighbour classifier; ANFIS: Adaptive neuro-fuzzy inference system; ANN: Artificial neural networks; ANTDE: Ant and differential evolution; ApEn: Approximate entropy; AR: Autoregressive; BP: Back-propagation; CA: Cultural algorithm; CP: Cerebral palsy; CrEn: Cumulative residual entropy; DBI: Davies-Bouldin index; DE: Differential evolution; DEFLDA: Differential evolution-fuzzy-linear discriminant analysis; EC: Evolutionary computing; ECOC: Error-correcting -output code; EEG: Electroencephalogram; ELM: Extreme learning machine; EMD: Empirical mode decomposition; EMG: Electromyogram; EOG: Electrooculogram; EP: Evolutionary programming; ES: Evolutionary strategy; FCM: Fuzzy C-means; FCNN: Fuzzy clustering neural network; FFT: Fast fourier transform; FGIHM: Fuzzy genetic implementation Hill-based muscle model; FGMM: Fuzzy Gaussian mixture model; FL: Fuzzy logic; FLDA: Fuzzy linear discriminant analysis; FLDI: Fishers linear discriminant index; FRRN: Fuzzy relational rule network; FSVM: Fuzzy support vector machine; FuzzyEn: Fuzzy entropy; FMMNN: Fuzzy Min-Max neural network; GA: Genetic algorithm; GBLS: Genetics-based learning system; GEP: Gene expression programming; GMM: Gaussian mixture models; GP: Genetic programming; HMM: Hidden Markov models; HSCS: Hybrid soft computing system; IMF: Intrinsic mode function; LDA: Linear discriminant analysis; LFP: Local field potential; LMS: Least mean square; MLP: Multilayer perceptions; mRMR: Minimum redundancy maximum relevance; MUAP: Motor unit action potential; NFA: Neuro-fuzzy agents; OAA: One-against-all; OAO: One-against-one; OLDA: Orthogonal linear discriminant analysis; PCA: Principal component analysis; PNN: Probabilistic neural network; PSO: Particle swarm optimization; PSOFLDA: Particle swarm-fuzzy-linear discriminant analysis; QDA: Quadratic discriminant analysis; RBF: Radial basis function; RFNN: Recurrent fuzzy neural network; RMS: Root mean square; RQA: Recurrence qualification analysis; SampEn: Sample entropy; SC: Soft computing; SCS: Supervisory control system; SI: Swarm intelligence; SOM: Self-organizing map; SVM: Support vector machine; ULDA: Uncorrelated discriminant analysis; VM: Virtual muscle.

## Competing interests

The authors declare that they have no competing interests.

## Authors’ contributions

HBX initiated the idea and draft the manuscript. GTR, SWB collected and analyzed the references, discussed the results, and revised the manuscript. SD supervised the overall project. All authors read and approved the final version of the manuscript.

## Authors’ information

HBX (PhD in Biomedical Engineering) is a Vice Chancellor’s Research Fellow in the Graduate School of Biomedical Engineering (GSBME), the University of New South Wales (UNSW). His research interests include neuromuscular bioelectronics and biomechanics, human computer interface, biomedical signal processing, and nonlinear time series analysis. GTR is currently a PhD candidate in GSBME, UNSW. His research interests include large-scale optimization of biophysical systems and modelling of excitable tissue. SWB (PhD in Biomedical Engineering) is currently a research associate in GSBME, UNSW. His research interests include modelling of biophysical systems and adaptive data processing of biomedical signals. SD (PhD in Biomedical Engineering) is an Associate Professor in GSBME, UNSW. His research has been multidisciplinary, involving techniques ranging from computational modelling and large-scale systems identification, through to electrophysiological measurements and mechanical testing.
